# PF4‐associated immune thrombocytopenia and thrombosis (PITT)—More than heparin and vaccines

**DOI:** 10.1111/bjh.20125

**Published:** 2025-04-28

**Authors:** Michael Makris

**Affiliations:** ^1^ School of Medicine and Population Health University of Sheffield Sheffield UK

**Keywords:** anti‐PF4, HIT, PITT, VITT

## Abstract

A young woman presented with portal vein thrombosis and thrombocytopenia and developed further extensive thrombosis following treatment with heparin. Despite no previous heparin exposure, she was found to be strongly positive for anti‐platelet factor 4 (PF4) antibodies. She had acute cytomegalovirus (CMV) infection and a monoclonal gammopathy.

Commentary on: Nicolson et al. Anti‐PF4 mediated thrombocytopenia and thrombosis associated with acute cytomegalovirus infection displays both HIT‐like and VITT‐like characteristics. Br J Haematol 2025; 206:1737‐1742.

In their paper, the authors describe a young woman with a low level of monoclonal gammopathy and acute CMV infection who presented with thrombocytopenia and thrombosis and who was found to have antibodies against PF4.^1^

Thrombocytopenia and thrombosis following heparin exposure (HIT) have been recognised for more than 50 years and are known to be due to anti‐PF4 antibodies. PF4 is a positively charged protein, and the binding to it by the strongly negatively charged heparin molecules induces a conformational change allowing antibody binding.

In 2008, it was shown that these anti‐PF4 antibodies could occur in the absence of heparin exposure in a condition termed spontaneous HIT.^2^ This condition, however, remained extremely rare, with only occasional case reports until early 2021. March 2021 saw an explosion of cases of thrombocytopenia and thrombosis following vaccination (VITT) with the Astra Zeneca and Johnson & Johnson adenoviral COVID‐19 vaccines.^3^ In the last couple of years, individuals with a condition very similar to VITT but without vaccination have been reported following adenoviral infections or in individuals with monoclonal gammopathy^4^; this created an issue because the V in VITT stands for vaccine‐associated, yet these persons had no recent history of vaccination. In view of this, the term PF4‐associated immune thrombocytopenia and thrombosis (PITT) would seem a more appropriate umbrella term for these similar disorders (Figure 1).

**FIGURE 1:**
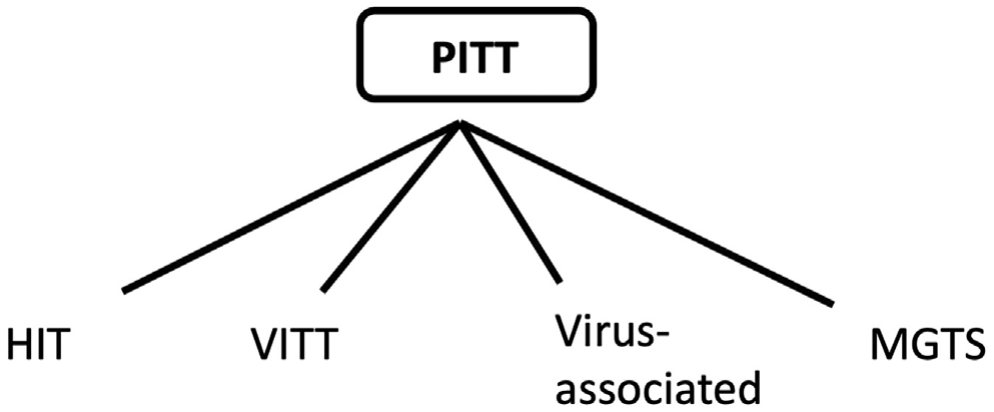
The PF4‐associated immune thrombocytopenia and thrombosis (PITT) disorders. HIT, Heparin‐induced thrombocytopenia; VITT, Vaccine‐induced immune thrombocytopenia and thrombosis; MGTS, Monoclonal gammopathy of thrombotic significance.

The case report in their paper concerns a young woman without a history of vaccination or heparin exposure, who was admitted to hospital with portal vein thrombosis and thrombocytopenia. Following treatment with heparin, she developed much more extensive thrombosis which led the authors to test her for anti‐PF4. Although the rapid chemiluminescence test (usually positive in HIT) was negative, she was strongly positive when tested with an ELISA test which is a classic feature of VITT.^5^ Further detailed testing revealed the presence of acute CMV infection on a background of a low level of monoclonal gammopathy. This monoclone was shown to be targeted against PF4. The epitopes on the PF4 molecule targeted in HIT (the equator) are different from those targeted in VITT (the poles) but importantly this patient had features of both. This is important because classical VITT can, at least in theory, be treated with heparin, while in this case this was clearly detrimental. It would seem sensible to avoid heparin in these PITT disorder patients unless detailed testing shows that heparin would not be platelet activating.

The authors suggest that any patients with the British Society for Haematology (BSH) Expert Haematology Panel criteria for VITT but without a history of vaccination should be tested for anti‐PF4 by an ELISA test. The criteria require a low level of fibrinogen and a high level of D‐Dimer, as well as the presence of thrombocytopenia and thrombosis.^3^ I would suggest that for now, just the presence of otherwise unexplained thrombocytopenia and thrombosis should be enough for anti‐PF4 ELISA testing. If future research shows that all cases of PITT have a low fibrinogen and high D‐Dimer level, then these can be included in the pretest requirements but to include them from now, it is perhaps premature.

Specialist platelet testing and even the anti‐PF4 ELISA test are not available in most hospitals, and even where available, they cannot be performed on a 24/7 basis. The rate of deterioration of these patients can be so rapid that treatment cannot usually wait for the results of these tests. In view of this, it would seem sensible to use non‐heparin anticoagulation and to institute treatment with intravenous immunoglobulin while waiting for the results. If a diagnosis of PITT is considered, testing for acute viral infections and immunoglobulin estimation for monoclonal gammopathy should be included.
